# Triade de Diamond: une manifestation exceptionnelle de la maladie de Basedow

**DOI:** 10.11604/pamj.2022.43.26.35441

**Published:** 2022-09-16

**Authors:** Kaoutar Rifai, Mohammed El Hassan Gharbi

**Affiliations:** 1 Service d´Endocrinologie et Maladies Métaboliques, Centre Hospitalo-Universitaire Ibn Sina, Faculté de Médecine et de Pharmacie, Université Mohammed V Souissi, Rabat, Maroc

**Keywords:** Triade de Diamond, maladie de Basedow, acropathie thyroïdienne, dermopathie thyroïdienne, exophtalmie, Diamond´s triad, Graves´ disease, thyroid acropathy, thyroid dermopathy, exophthalmos

## Abstract

Extra-ocular manifestations of Graves´ disease are rare and little known. We here report one of its manifestations: Diamond´s triad is defined as the association of exophthalmos, acropachy (<1%) and pretibial myxedema (1.5-2%). It has been rarely found in patients with Graves´ disease. We here report the case of a 33-year-old patient with no particular previous history, on follow-up for Graves´ disease, complicated by cardiac arrhythmia (atrial fibrillation), treated with synthetic antithyroid drugs followed by total thyroidectomy after cardiac and thyroid stabilization. The patient reported purplish lesions on the anterior face of the legs that had persisted for several years. Clinical examination showed voluminous vascular goiter and vitiligo lesions, associated with bilateral exophthalmos, with Clinical Activity Score < 3 (A) (amended by European Group on Graves' orbitopathy (EUGOGO)), discrete pretibial oedema with purple erythematous patches, painless and firm to palpation, on the anterior face of the legs (B) and pretibial myxedema suggesting pretibial dermopathy (Basedow´s disease). The fingers and toes are clubbed in the shape of a drumstick (digital hippocratism) (C, D), suggesting dysthyroid acropathy. Dysthyroid acropathy occurs in less than 1% of patients with Graves´ disease but is constantly associated with exophthalmos and pretibial myxedema, thus forming the Diamond Triad, as in the case of our patient.

## Image en médecine

Les atteintes extra-ophtalmologiques de la maladie de Basedow sont rares et peu connues, nous rapportons ici une de ses manifestations: la triade de Diamond qui se définit par une association, rarement retrouvé chez les patients avec maladie de Basedow, d´une exophtalmie avec une acropathie (<1%) et un myxœdème prétibial (1,5-2%). Il s´agit d´un patient âgé de 33 ans, sans antécédent notable, suivi pour une maladie de Basedow, compliquée d´une cardiothyréose avec une arythmie complète par fibrillation auriculaire, traitée par antithyroïdiens de synthèse puis thyroïdectomie totale après stabilisation de l´état cardiaque et thyroïdien. Le patient rapportait des lésions à type de colorations violacées persistantes des faces antérieures des jambes évoluant depuis plusieurs années. À l´examen clinique, en dehors d´un volumineux goitre vasculaire et des lésions de vitiligo, nous notons une exophtalmie bilatérale avec un score EUGOGO d´activité clinique < à 3 (A), des œdèmes prétibiaux discrets avec des plaques érythémateuses violacées, indolores et fermes à la palpation située à la face antérieure des jambes (B) en rapport avec un myxœdème prétibial signant probablement la dermopathie prétibiale de la maladie de Basedow. Les doigts et les orteils sont boudinés en forme de baguette de tambour réalisant ainsi un hippocratisme digital (C, D) rentrant dans le cadre de l´acropathie dysthyroïdienne. Cette dernière est présente dans moins de 1% de la maladie de Basedow mais elle est constamment associée à une exophtalmie et à un myxœdème prétibial formant ainsi la triade de Diamond, comme c´était le cas pour notre patient.

**Figure 1:**
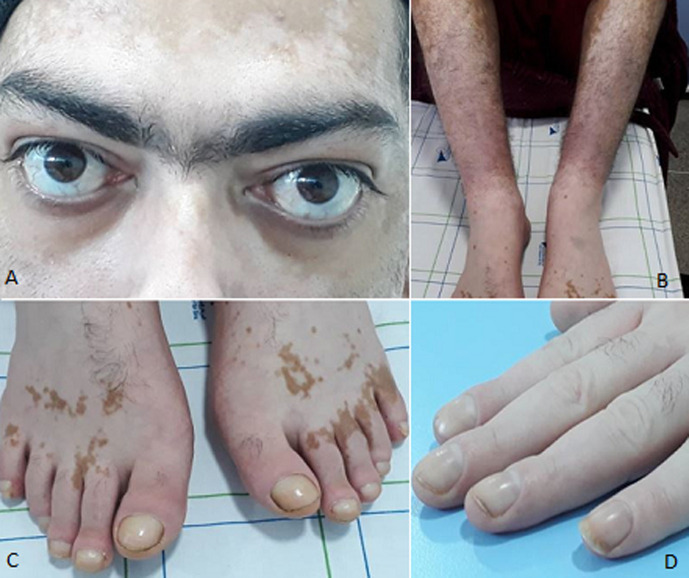
A) exophtalmie bilatérale en rapport avec la maladie de Basedow, B) myxœdème prétibial, C) hippocratisme digital au niveau des pieds en rapport avec l´acropathie, D) hippocratisme digital au niveau des mains en rapport avec l´acropathie

